# Next-Generation Flap Monitoring: Systematic Review and Meta-Analysis of Hyperspectral Imaging

**DOI:** 10.1055/a-2660-4344

**Published:** 2026-01-30

**Authors:** Parintosa Atmodiwirjo, Cristabella Rininta

**Affiliations:** 1 Division of Plastic Reconstructive and Aesthetic Surgery, Department of Surgery, Dr. Cipto Mangunkusumo National Referral Hospital, Faculty of Medicine, University of Indonesia, Jakarta, Indonesia

**Keywords:** hyperspectral imaging, flap monitoring, flap viability, systematic review and meta-analysis

## Abstract

Early detection of perfusion deficits is crucial for optimal flap outcomes. Hyperspectral imaging (HSI) offers a non-invasive method to assess tissue perfusion, potentially detecting complications earlier than Doppler ultrasound or near-infrared spectroscopy. This systematic review and meta-analysis evaluate the efficacy and diagnostic accuracy of HSI in monitoring flap viability, particularly in detecting ischemia and necrosis. A systematic search of PubMed, ScienceDirect, and Cochrane Library was conducted following PRISMA 2020 guidelines. Studies on HSI's role in flap viability were critically appraised using the Critical Appraisal Skills Programme checklist. A random-effects model was applied for the meta-analysis. Nine studies were included, six focusing on flap complications. HSI demonstrated a median sensitivity of 93% (63–100%) and a median specificity of 96% (81–100%) for detecting compromised flaps, outperforming clinical assessments in some cases. Significant differences were observed between viable and necrotic tissues in four key HSI parameters: Oxygen saturation, tissue hemoglobin index (THI), tissue water index, and near-infrared perfusion index (NIR-PI). THI and NIR-PI effectively differentiated venous congestion from arterial occlusion. However, heterogeneity across the studies indicated a need for standardized protocols. Notably, HSI detected perfusion deficits up to 4.8 hours before clinical signs. HSI shows promise for postoperative flap monitoring, enabling earlier detection of ischemia and necrosis. Future research should focus on standardized imaging protocols, real-time analysis, and larger multicenter trials to confirm HSI's clinical utility and cost-effectiveness.

## Introduction

Flap surgery is a cornerstone of reconstructive procedures, enabling the restoration of form and function following trauma, oncologic resection, or congenital anomalies. Despite advances in surgical techniques and perioperative care, the success of flap procedures hinges on ensuring adequate blood supply to the transplanted tissue. Compromised perfusion remains one of the most significant challenges, leading to complications such as partial or total flap necrosis, infection, and surgical failure. Even with reported flap survival rates up to 95%, 1 complications underline the critical need for effective methods to monitor flap viability during the perioperative period.

Traditionally, the assessment of flap perfusion has relied on clinical examination and adjunctive imaging modalities, including Doppler ultrasound, near-infrared spectroscopy (NIRS), and indocyanine green (ICG) angiography. While these methods have demonstrated utility, they often fall short in providing real-time, objective, and detailed spatial information about tissue oxygenation and perfusion. This limitation is particularly pronounced in complex reconstructive cases, where early detection of ischemia is crucial for timely intervention and flap salvage.

Hyperspectral imaging (HSI) is a novel, non-contact imaging modality that combines digital imaging with high-resolution spectroscopy. It operates by illuminating tissue with a calibrated white light source and capturing the reflected light across a broad spectral range (typically 500–1,000 nm), producing datasets containing both spatial and continuous spectral information. The reflected light is analyzed to extract physiologically relevant parameters such as tissue oxygen saturation (StO _2_ ), which reflects superficial microcirculatory oxygenation (tissue measurement depth up to ∼1 mm); tissue hemoglobin index (THI), which indicates the total hemoglobin content; near-infrared perfusion index (NIR-PI), which provides perfusion data from deeper layers (∼4–6 mm); and tissue water index (TWI), which assesses hydration and edema.

**Fig. 1:**
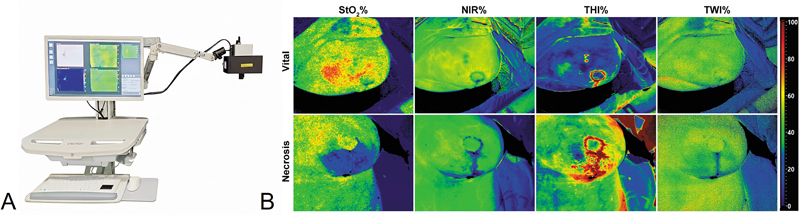
( **A** ) TIVITA® Tissue hyperspectral imaging system (wheeled base not shown); adapted from Pachyn et al. (2024). 15 ( **B** ) HSI parameter maps used in flap monitoring; adapted from Pruimboom et al. (2022). 11 HSI, hyperspectral imaging; NIR-PI, near-infrared perfusion index; StO _2_ , oxygen saturation; THI, tissue hemoglobin index.

These measurements are processed and displayed as color-coded parameter maps, enabling clinicians to assess regional perfusion and viability in flaps without needing contrast agents or direct contact with the tissue. A representative image of a clinically used HSI system (TIVITA® Tissue), along with examples of color-coded HSI parameter sets (StO _2_ , NIR, THI, TWI), for a vital and a necrotic mastectomy skin flap, with perfusion deficits clearly visualized in the necrotic areas, is shown in Fig. 1 .

Because HSI relies on a calibrated, self-contained LED light source, stable illumination is essential. In routine use, the camera is held 50 cm from the tissue being monitored, for example, a flap. One scan of the tissue typically takes less than 10 seconds. Ambient lighting—sunlight and ambient light—needs to be suppressed by darkening the room, as light fluctuations will distort the measurements.

Over the past decade, HSI has been increasingly explored in various surgical disciplines, including reconstructive, maxillofacial, and plastic surgery. Preliminary studies suggest that HSI can detect perfusion deficits earlier than clinical assessments, allowing for timely interventions that may improve outcomes. However, despite its potential, HSI is not yet widely adopted in clinical practice, partly due to challenges in standardizing imaging protocols, interpreting data, and integrating the technology into surgical workflows.

This systematic review and meta-analysis aim to evaluate the efficacy and diagnostic accuracy of HSI in predicting flap survival and identifying complications such as ischemia and necrosis. By synthesizing evidence from current literature, we seek to provide a comprehensive assessment of HSI's clinical utility, identify gaps in knowledge, and propose future directions for research and implementation.

## Methods

**Fig. 2:**
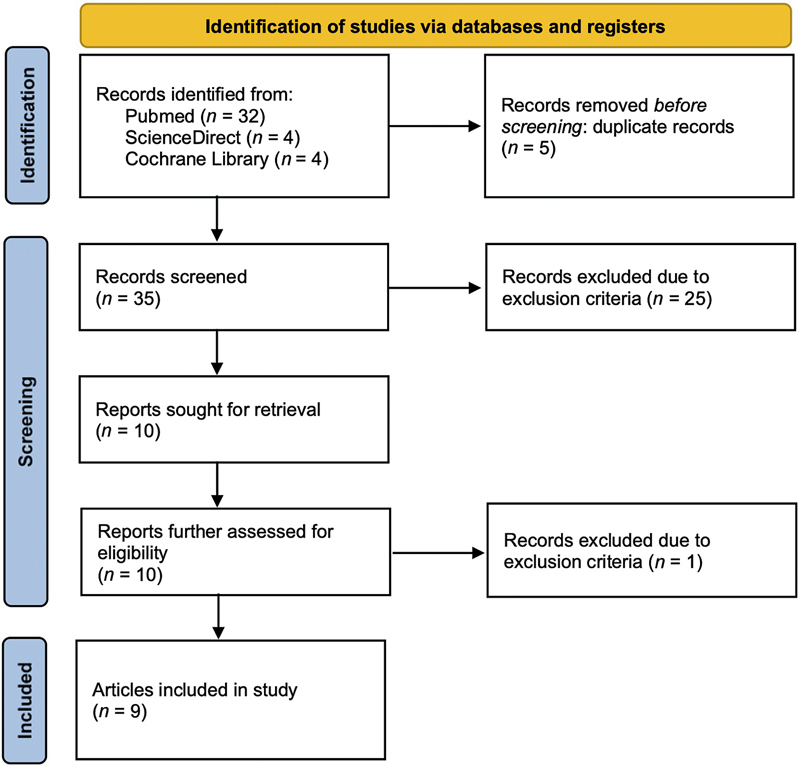
PRISMA flow diagram for this study. PRISMA, Preferred Reporting Items for Systematic Reviews and Meta-Analyses.

A systematic search was conducted in October 2024 through PubMed, ScienceDirect, and Cochrane Library according to the Preferred Reporting Items for Systematic Reviews and Meta-Analyses (PRISMA 2020) statement. 2 The search terms “hyperspectral,” “imaging,” and “flap” were utilized, with slight adjustments to suit each database's search functions. Inclusion and exclusion criteria are listed in Table 1 . The studies were first screened for duplicates; subsequently, articles were excluded based on the analysis of the title, abstracts, and full text, respectively. The PRISMA workflow is shown in Fig. 2 ; the study was registered with the International Platform of Registered Systematic Review and Meta-analysis Protocols (INPLASY 3 ) under protocol number INPLASY2024120111.

**Table 1:** Inclusion and exclusion criteria

Inclusion criteria
Randomized controlled trials, cohort studies
Postoperative free-flap evaluation using HSI
Articles published in a Scopus-indexed journal
Exclusion criteria
Case series, case reports, thesis, and gray literature
Non-human studies

Abbreviation: HSI, hyperspectral imaging.

Critical appraisal of the studies included was assessed using the Checklist of the Critical Appraisal Skills Program Diagnostic test 2024. 4 Meta-analysis of HSI parameters was performed using the online software tool MetaAnalysisOnline.com. 5 OpenAI's ChatGPT (2024, 2025) was used to support language refinement.

## Results

There are nine studies included in this review (see Table 2 ). All studies can be described as observational cohort studies; three were retrospective and six were prospective by design. Of these, three studies were excluded from the quantitative meta-analysis section on diagnostic accuracy and HSI parameters 6 7 8 as no complications of the flap surgeries were reported in these.

**Table 2:** Study characteristics

Study	Study design	Postoperative evaluation	Flap survival	Partial necrosis	Other complications	Sample size	Flap type	Outcome summary	Study limitations
Felicio-Briegel et al. (2024) 6	Prospective	HSI performed intraoperatively and on days 1, 2, 3–6, 7–9, 10–11, 12–15 postop	No flap loss occurred	None reported	2 patients had wound dehiscence requiring revision	14 patients	Radial forearm free flaps, placed intraorally	HSI showed feasibility for monitoring free flaps and matched clinical findings, but illumination and visualization were challenging	Small sample size; technical challenges with imaging
Kohler et al. (2021) 13	Prospective	HSI at *t*_0_ (day of surgery), *t*_1_ (16–28 hours postop), *t*_2_ (39–77 hours postop), plus clinical and Doppler checks	4 flaps lost (18.2%), 2 partial revisions	2 cases of partial flap loss (9.1%)	1 venous thrombus in total flap losses	22 flaps (16 regular healing, 6 with full (4) or partial (2) revisions)	ALT, latissimus dorsi, DIEP, MS2-TRAM, rectus abdominis	HSI detected perfusion issues earlier than clinical/Doppler, with StO _2_ and NIR below 40, indicating flap revision	Sample size; endpoint heterogeneity
Merdasa et al. (2023) 7	Prospective	HSI performed only ex vivo, immediately postdissection	N/A	N/A	No complications noted during imaging	8 patients	Forehead flap (for brow lift)	HSI accurately mapped sO _2_ levels, showing a decrease from the flap base to tip and a rapid drop after flap excision	Small sample size; limited to excision phase
Pruimboom et al. (2022) 11	Prospective	HSI and Doppler checks every hour for 12 hours, then daily for 3 days	No DIEP flap necrosis	Mastectomy skin flap necrosis (MSFN) in 3 of 10 patients, detected by decreased oxygenation	1 reoperation due to MSFN; 2 recovered conservatively	10 patients (3 with necrosis, 7 without)	DIEP flap	HSI detected necrosis early; significant difference in StO _2_ % between vital and necrotic flaps	Small sample size; focus on immediate flap viability
Schulz et al. (2020) 14	Retrospective	HSI used on days 1–7	One flap lost to venous stasis	Partial necrosis in 15/16 cases	1 total flap loss; minor wound complications in 15 cases	16 flaps (15 with partial necrosis, 1 full loss)	Latissimus dorsi, radialis, gastrocnemius, suralis, etc.	HSI detected perfusion issues early, helping with necrosis and venous stasis monitoring	Small sample size; no detailed analysis on wound dehiscence
Schulz et al. (2021) 12	Prospective	Postoperative evaluation was done daily for 7 days using HSI; distinction between arterial occlusion and venous congestion	One flap loss due to arterial occlusion	Nine cases of partial necrosis with locally impaired perfusion	One arterial occlusion requiring revision; flap not salvaged	17 flaps after 48 hours (8 vital, 9 partial necrosis); 1 arterial congestion case only at start	Anterolateral thigh and latissimus dorsi flaps	HSI effectively identified areas of impaired perfusion and provided guidance for flap monitoring and revision decisions	Sample size; high patient heterogeneity
Schulz et al. (2023) 9	Retrospective	HSI at 0–72 hours postop in three groups (≤24, 24–48, 48–72 hours); clinical and HSI assessments compared	84% flap success rate; 7 flaps lost (16%) due to venous congestion	N/A	7 flap losses due to venous congestion and venous thrombosis	42 flaps (35 vital, 7 avital) at *t*_0_ , 41 flaps (35 vital, 6 avital) at 48–72 hours)	ALT, LDM, DIEP, rectus abdominis	HSI detected venous congestion earlier than clinical assessments with higher sensitivity (63%) within 72 hours postop	Sample size; THI threshold probably not adequate; short monitoring window; missing data about flaps with arterial occlusion
Thiem et al. (2021) 10	Prospective	HSI and clinical assessments compared at *t*_0_ – *t*_10_	Seven complete flap failures; overall salvage rate was 63%	Not explicitly mentioned, but 19 flaps required re-exploration due to perfusion issues	Venous and arterial thrombosis, hematoma, and kinking of the pedicle were noted	65 flaps (46 no revision, 19 with revision)	Radial, ulnar forearm, osteocutaneous fibula, latissimus dorsi, scapula, and upper arm flaps	HSI detected malperfusion 4.8 hours earlier than clinical assessments, enhancing early detection and intervention for flap salvage	No randomization
Thoenissen et al. (2023) 8	Retrospective	HSI performed at intervals from 3 to 100 hours postop	No flap loss was observed during the study period	N/A	None reported	13 patients with free flap reconstructions, 10 controls	Free flaps for craniomaxillofacial reconstruction	HSI successfully measured flap parameters, showing a reduction in oxygenation but no flap loss stable imaging conditions are crucial	Small sample size; limited follow-up

Abbreviations: ALT, anterolateral thigh; DIEP, deep inferior epigastric perforator; HSI, hyperspectral imaging; LDM, latissimus dorsi; NIR, near-infrared perfusion index; StO _2_ , oxygen saturation; THI, tissue hemoglobin index.

**Fig. 3:**
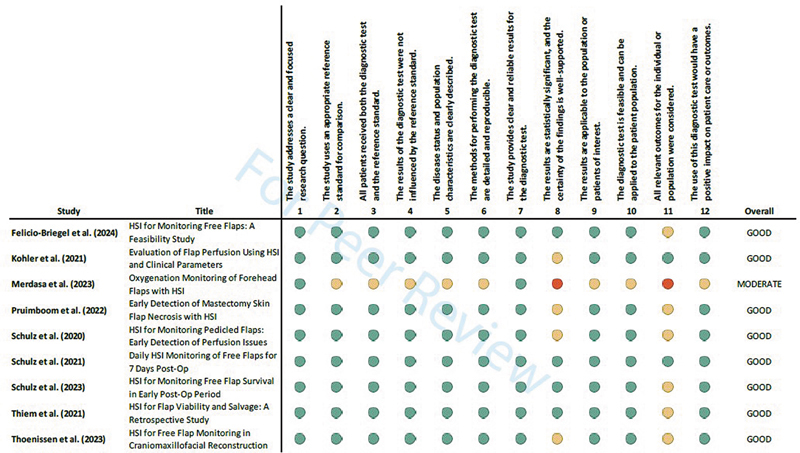
Critical appraisal using the Diagnostic Test Checklist of the Critical Appraisal Skills Programme 2024 (CASP). HSI, hyperspectral imaging.

The critical appraisal (see Fig. 3 ) showed moderate to good overall assessments, with one exception (6), scoring lower in almost all categories than the other studies, for the purpose of this analysis.

### Qualitative Summaries of the Studies

The application of HSI for evaluating flap survival has been extensively explored in these studies, highlighting its potential as a non-invasive, quantitative tool for monitoring microcirculation and detecting early perfusion compromise. HSI demonstrated promising diagnostic capabilities, although challenges related to standardization and clinical integration persist.

Thoenissen et al. 8 evaluated HSI in craniomaxillofacial reconstruction, focusing on key parameters such as oxygenation (StO _2_ %), THI, and TWI. Stable intrapatient parameters postoperatively and the absence of flap loss confirmed HSI's reliability under controlled conditions. However, the study highlighted significant limitations in intraoral imaging due to restricted access, raising concerns about its applicability in specific anatomical sites. Similarly, Schulz et al. 9 investigated HSI's diagnostic accuracy for extremity flaps and identified a THI threshold for detecting venous congestion. HSI proved more sensitive than clinical examination within the first 24 hours but showed no significant advantage over traditional methods by 72 hours, suggesting the need for optimized threshold calibration.

Thiem et al. 10 demonstrated HSI's ability to detect perfusion compromise earlier than clinical assessment in maxillofacial surgery. This time-sensitive advantage supports its value in high-risk postoperative monitoring, although operator dependency and inconsistent imaging conditions posed barriers to broader implementation. In breast reconstruction, Pruimboom et al. 11 piloted the use of HSI in deep inferior epigastric perforator (DIEP) flaps. The study found a correlation between decreased StO _2_ % and flap necrosis, proposing a cutoff value for intervention. However, the small sample size and lack of interventional validation limit the study's generalizability.

Additional insights came from Schulz et al., 12 who evaluated THI and TWI as predictive markers of flap survival. Their ability to distinguish between viable, compromised, and necrotic tissues bolsters HSI's diagnostic potential. However, variability in light conditions during data acquisition raises concerns about reproducibility across clinical settings. Kohler et al. 13 further supported HSI's utility in detecting ischemic changes in soft tissue flaps. Their proof-of-concept study proposed critical thresholds for intervention, but the lack of large-scale validation diminishes its immediate clinical applicability.

In the context of intraoral reconstruction, Felicio-Briegel et al. 6 confirmed the feasibility of HSI for monitoring radial forearm free flaps (RFFs). While no flap loss was reported in their cohort, the study highlighted challenges such as light interference and access difficulties that hinder consistent data acquisition. Meanwhile, Merdasa et al. 7 advanced HSI methodology by integrating spectral unmixing to map oxygen saturation in forehead flaps. By accounting for multiple chromophores, their approach enhanced measurement accuracy but remains complex for routine clinical use. Earlier studies by Schulz et al. 14 laid foundational benchmarks by addressing light control and standardizing imaging distances, which remain critical for achieving consistent results. The three studies by Schulz et al. (2020, 14 2021, 12 and 2023 9 ) were conducted at the same institution and within overlapping study times, and hence may potentially include overlapping patient cases, although this was not explicitly stated in the publications.

Collectively, these studies underscore HSI's transformative potential in flap survival evaluation. Its ability to provide real-time, quantitative insights into tissue perfusion positions it as a valuable adjunct to traditional monitoring techniques. All of the included studies employed a TIVITA® (more precisely, TIVITA® Tissue, where specified) HSI system (successor model: TIVITA® 2.0) from the manufacturer Diaspective Vision GmbH (Germany), which was acquired by KARL STORZ SE & Co. KG (Germany) in 2025. The only exception was the study by Merdasa et al., 7 which utilized a highly customized HSI setup based on a HySpex camera from Norsk Elektro Optikk AS (Norway). To the authors' knowledge, the current TIVITA® models are the only HSI systems registered under the European Union Medical Device Regulation, and no HSI systems are currently registered as medical devices with the U.S. FDA.

Study limitations such as small sample sizes, protocol variability, and operator dependency highlight the need for standardized methodologies and larger multicenter trials to validate their clinical utility. With further development, HSI could significantly enhance outcomes by enabling timely detection and intervention in cases of flap compromise.

### Diagnostic Accuracy of Hyperspectral Imaging

For five studies, relevant numbers of favorable and unfavorable clinical outcomes of flap surgeries were reported, and diagnostic accuracy values of HSI could be deducted (see Table 3 ). Schulz et al. 14 were excluded here, as the authors explicitly excluded analytical statistics due to the small sample size of 15 flaps.

**Table 3:** Diagnostic accuracy analysis of hyperspectral imaging

Study	Sensitivity	Specificity	PPV	NPV	Thresholds	Comments
Kohler et al. (2021) 13	100%	100%	100%	100%	StO _2_ <40% and NIR <40%	HSI versus clinical evaluation plus Doppler US; here, at *t*_2_ = second day postoperatively. Note: HSI could detect malperfusion earlier than standard procedure.
Pruimboom et al. (2022) 11	93%	81%	68%	96%	StO _2_ <36.3%	HSI versus clinical appearance; here, picked from a reported ROC graph, for high sensitivity and acceptable specificity.
Schulz et al. (2021) 12	92%	93%	80%	97%	THI ≥53%	HSI versus clinical appearance; further threshold values proposed, but with lower AUC values than THI: NIR ≤25%, TWI ≤43%, StO _2_ ≤22%.
Schulz et al. (2023) 9	63%	96%	75%	94%	THI ≥53%	HSI versus clinical outcome; clinical evaluation had 63% sensitivity and 100% specificity
Thiem et al. (2021) 10	100%	100%	100%	100%	StO _2_ ≤32% OR StO _2_ difference > − 38% OR NIR ≤32.9 OR NIR difference ≥ −13.4%	HSI versus clinical outcome; clinical evaluation was used for final decision making, and had also 100% sensitivity and specificity, but HSI could detect malperfusion 4.8 hours earlier

Abbreviations: HSI, hyperspectral imaging; NIR, near-infrared perfusion index; StO _2_ , oxygen saturation; THI, tissue hemoglobin index; TWI, tissue water index.

None of the studies relied on HSI for clinical decision-making, for example, flap revision, even if HSI indicated flap complications earlier than experts' observations (e.g., in Thiem et al. 10 ), due to the novelty of HSI, according to the authors.

The HSI variables and threshold values employed varied across the studies, ranging from StO _2_ or THI alone to combinations of StO _2_ and NIR or StO _2_ and NIR and their differences over time.

HSI threshold values for determining diagnostic values were calculated ex-post in the studies, with the exception of Schulz et al., 9 which assumed a threshold value of THI ≥53%, based on the previous study by Schulz et al., 14 but the HSI values were matched retrospectively from the clinic's database.

The reference method differed across the studies, as diagnostic accuracy values for HSI were either measured against the actual clinical outcome or clinical evaluation. Two studies reported diagnostic accuracy for HSI and for clinical evaluation versus clinical outcomes; in both studies, 9 10 HSI measurements indicated earlier signs of flap complications. In Thiem et al., 10 sensitivity and specificity were 100% versus clinical evaluation. Schulz et al. 9 also reported sensitivity and specificity for all three given time points; although HSI yielded relatively low diagnostic values for all time points taken together (63% sensitivity, 96% specificity), it was still comparable to clinical evaluation (63% sensitivity, 100% specificity). The other three studies that measured HSI versus the clinical outcomes had sensitivity of 92%, 93%, and 100%, and specificity of 93%, 81%, and 100%, respectively. 11 12 13 For Pruimboom et al., 11 in particular, diagnostic values were not reported directly, but were deducted from a reported Receiver operating characteristic (ROC) graph of a simple logistic regression of StO2% (necrotic versus vital), and picked for high sensitivity, and acceptable specificity. Across all these five studies, median sensitivity was 93%, and specificity was 96%.

### Hyperspectral Imaging Values for Vital versus Necrotic Flaps

Six studies provided quantitative HSI parameter data for necrotic versus viable tissue, revealing significantly different values for THI, TWI, and NIR in most. StO _2_ showed high variability across time points, suggesting a need for standardized imaging protocols. Meta-analysis indicated that THI tends to be higher under venous congestion, while NIR is usually lower in necrotic regions, reflecting reduced tissue perfusion. Kohler et al. 13 only reported values for StO _2_ and NIR, while the other studies also included THI and TWI. A total of 58 flaps in the necrotic groups and 127 flaps in the vital groups were analyzed (without Kohler et al., 13 52 and 111 flaps, respectively), while for Schulz et al., 14 all 15 flaps were counted in both groups, since all were vital, but had also necrotic wound margins that were measured. The plastic surgeries conducted and the flap origins were highly diverse within and across studies, contributing to considerable heterogeneity.

The studies did not identify or report explicit cases of arterial thrombosis—typically a significantly rarer complication in comparison to venous congestion—with the exception of Schulz et al. 12 In the latter study, one case of arterial occlusion was reported, requiring immediate revision at the earliest time point, with StO _2_ and THI at only 3%; this one case was not considered for this analysis.

The six studies analyzed reported highly varying time points for HSI measurements. To allow for better comparison across the studies, a consensus time of 48 hours postoperatively was chosen; alternatively, the second day postoperatively, or, if not reported as a precise time point, as 48 to 72 hours postoperatively. Only Schulz et al. 9 presented a full table with all HSI values for all time points; for all other studies, values were extracted from the graphs and accompanying data provided. For Schulz et al., 14 standard deviation (SD) values at 48 hours were not given; therefore, the highest SD reported for each group was considered instead. Furthermore, the monitor island, which stayed vital in this study across the cases analyzed, was chosen for analysis. In Pruimboom et al., 11 which focused on mastectomy flap surgeries, the area analyzed was at the “vertical scar underneath the skin island of the DIEP ﬂap to the inframammary fold.” In the following, a meta-analysis for each of the four HSI parameters, StO2, THI, NIR, and TWI, is shown.

**Fig. 4:**
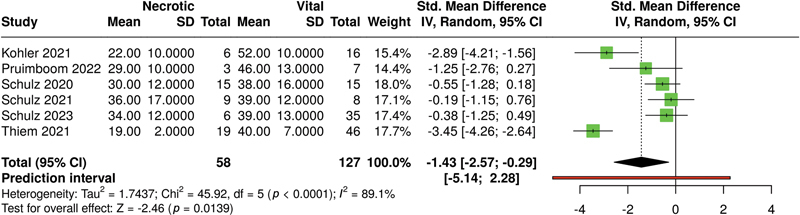
Meta-analysis of StO _2_ levels of HSI associated with necrotic versus vital tissue at ca. 48 hours postoperatively. HSI, hyperspectral imaging; StO _2_ , oxygen saturation.

For the parameter StO _2_ (see Fig. 4 ), the analysis performed used a random-effects model with inverse variance method to compare the standardized mean difference, and the *p* -value smaller than 0.05 implies that StO _2_ being lower in the necrotic group is of a significant statistical difference.

A significant heterogeneity was detected ( *p* < 0.01), suggesting inconsistent effects in magnitude and/or direction. The *I* ^2^ value indicates that 89% of the variability among studies arises from heterogeneity rather than random chance.

**Fig. 5:**
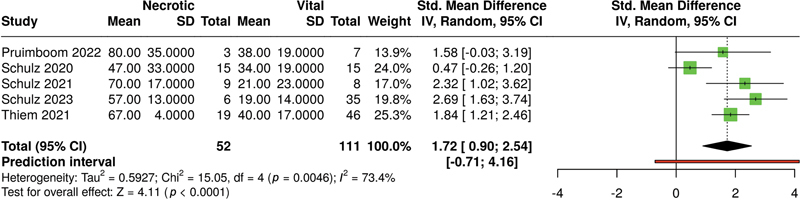
Meta-analysis of THI levels of HSI associated with necrotic versus vital tissue at ca. 48 hours postoperatively. HSI, hyperspectral imaging; THI, tissue hemoglobin index.

For the parameter THI (see Fig. 5 ), necrotic flaps showed, overall, significantly higher THI values than vital flaps ( *p* < 0.01 in a random-effects model). Of note, only complications due to venous congestion were reported here; for arterial occlusion, the literature showed very low THI values. Heterogeneity was significant ( *p* < 0.01), with an *I* ^2^ value of 73%.

**Fig. 6:**
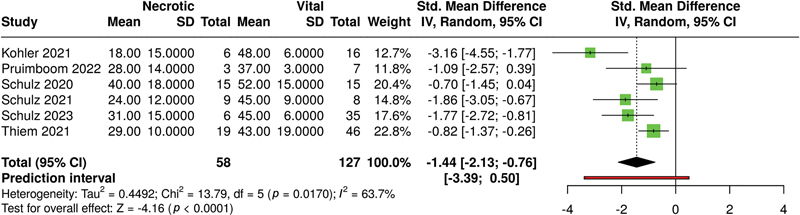
Meta-analysis of NIR levels of HSI associated with necrotic versus vital tissue at ca. 48 hours postoperatively. HSI, hyperspectral imaging; NIR-PI, near-infrared perfusion index.

The overall effect for NIR (see Fig. 6 ) was significant ( *p* < 0.01 in a random-effects model); necrotic flaps have lower NIR values than vital flaps. Heterogeneity for NIR was significant ( *p* < 0.05), and *I* ^2^ was 64%.

**Fig. 7:**
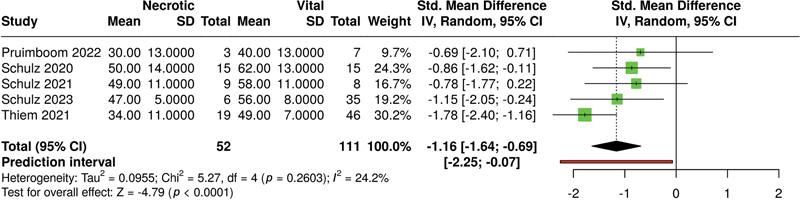
Meta-analysis of TWI levels of HSI associated with necrotic versus vital tissue at ca. 48 hours postoperatively. HSI, hyperspectral imaging; TWI, tissue water index.

TWI values (see Fig. 7 ) differed significantly for necrotic versus vital flaps overall ( *p* < 0.01 in a random-effects model), being lower for necrotic flaps. Notable variability was not detected, indicating that the effect sizes across studies were uniform in both magnitude and direction; *I* ^2^ was 24%, indicating consistent results for TWI across studies.

## Discussion

This systematic review and meta-analysis confirm the potential of HSI as a transformative tool in flap monitoring, enabling early detection of perfusion deficits and offering valuable insights into tissue viability. By synthesizing evidence from nine studies, we analyzed the capability of the four core parameters of HSI, StO _2_ , THI, TWI, and NIR for distinguishing viable from compromised flaps. The diagnostic accuracy reported across the included studies consistently highlighted the sensitivity and specificity of HSI in identifying venous congestion and arterial occlusion before clinical signs became apparent. Our findings support the utility of HSI as a non-invasive, quantitative adjunct to traditional flap monitoring techniques.

The meta-analysis revealed statistically significant differences in HSI parameters between viable and necrotic tissues for all four parameters. THI and NIR showed opposite trends for venous and arterial complications, making them valuable markers for early intervention. The ability of HSI to detect perfusion deficits several hours earlier than clinical evaluation, as shown in studies like Thiem et al., 10 where compromised perfusion was identified up to 4.8 hours before changes in flap color, turgor, or capillary refill were noted. In Kohler et al., 13 HSI was able to detect perfusion deficits in all flaps requiring revision before abnormalities were observed either clinically or via Doppler, particularly at the 16- to 28-hour postoperative mark ( *t*_1_ ). This suggests that while HSI is not a continuous monitoring tool, its high spatial resolution and multiparametric output can provide earlier or more sensitive detection of malperfusion than Doppler in certain postoperative settings, especially for flaps without visible skin islands. However, in other circumstances, and especially in an intraoperative setting, real-time perfusion monitoring technologies like Doppler ultrasound and NIRS may be more practical and actionable.

Despite these strengths, the significant heterogeneity of effect sizes for the HSI parameters across the studies, with the exception of TWI, highlights the need for standardized protocols and threshold values. The variability was likely further exacerbated by differences in surgery types, imaging timing, operator usage, systems, and settings used.

Notably, HSI demonstrated a median sensitivity of 93% across the five studies analyzed for this, with a range of 63 to 100%, and a median specificity of 96% (81–100%) in detecting flap complications, outperforming clinical assessments in some cases. However, its diagnostic utility varied with the choice of parameters and varying cutoff thresholds, indicating the need for tailored protocols depending on the type of flap and surgical context. Further limitations of this analysis are that the studies applied the threshold levels ex-post; the time point, or time frame for analyzing the accuracy, differed across studies, further complicating the comparability across studies.

Another critical limitation pertains to the heterogeneity of flap types included across the studies reviewed. Given that HSI relies on light reflectance, tissue characteristics—such as flap thickness, vascular architecture, and anatomical location—can significantly influence perfusion assessment. Thinner flaps like RFFs or forehead flaps may allow more accurate surface-level oxygenation mapping, while bulkier flaps such as latissimus dorsi, rectus abdominis, or anterolateral thigh flaps may challenge HSI's limited penetration depth. Additionally, the surgical context ranged from intraoral and craniomaxillofacial reconstructions to breast and extremity reconstructions, further adding variability. These differences likely contribute to the observed heterogeneity in HSI thresholds and diagnostic performance. Future studies should stratify flap types by tissue composition and anatomical site to develop more tailored HSI parameter cutoffs and improve the generalizability of findings.

An additional consideration that merits further research is the role of skin pigmentation, and in turn, also tattoos, on HSI measurement capability and standardization, as a recent study pointed out challenges with HSI performance for patients with darker skin tones. 15 According to the main medical HSI system manufacturer, the HSI parameter TWI should not be influenced by skin pigmentation, StO _2_ might be affected, and THI and NIR to a certain extent. 16

When comparing HSI with NIRS, as reviewed by Lindelauf et al., 17 our findings extend the understanding of HSI as a non-invasive monitoring tool. While NIRS offers continuous monitoring with relatively straightforward implementation, its dependency on sensor placement limits its application in certain flap types, such as intraoral or buried flaps. HSI, being contactless, avoids these limitations and provides spatially resolved imaging, making it suitable for diverse reconstructive scenarios. The review emphasized the high flap survival rates associated with NIRS (99.2% compared with HSI's 92.5%) but highlighted the limited penetration depth and variable algorithmic accuracy of NIRS devices. In contrast, HSI's broader spectrum of parameters enables a more nuanced analysis of microcirculation, potentially identifying both arterial and venous issues with greater precision. Additionally, while Lindelauf et al. 17 noted the scarcity of HSI studies ( *n* = 5) compared with NIRS ( *n* = 16), this review bridges this gap by including more recent HSI-focused research, thereby providing a robust evidence base for its clinical utility.

When compared with other technologies for flap perfusion monitoring, including indocyanine green fluorescence angiography (ICG-FA) and computed tomography perfusion imaging, HSI exhibits unique advantages while addressing some of their limitations.

ICG-FA is a widely adopted technique for intraoperative and postoperative flap monitoring, providing real-time visualization of tissue perfusion. The use of ICG has demonstrated its utility in reducing complications, as highlighted in a study by Fadell et al., 18 which underscores its critical role in assessing flap viability. However, challenges persist, including the need for precise dosing, variability in fluorescence interpretation, and limited penetration depth, which may affect accuracy in thicker tissues. A recent study 19 further refined the understanding of ICG-FA's application in flap perfusion monitoring, by evaluating fluorescence intensity between standard and diluted doses of ICG, concluding that diluted doses maintained diagnostic efficacy while reducing potential adverse effects and cost.

In contrast, HSI does not require contrast agents, eliminating the potential risks of allergic reactions or nephrotoxicity associated with ICG. Additionally, HSI offers a broader spectral range, enabling detailed analysis of tissue oxygenation, hemoglobin distribution, and water content, which are critical parameters in assessing flap viability.

Further flap monitoring techniques are reviewed elsewhere, 20 including 11 more commonly used techniques, among them Handheld Doppler and Color Duplex Ultrasonography, and six experimental techniques, including HSI. It is noted that HSI is still a relatively new method, which the authors connoted having only “low” efficacy, without giving specific quantitative context; with this study, we hope that the diagnostic accuracy can be re-evaluated.

This review offers a systematic inclusion of both quantitative and qualitative analyses of HSI parameters, providing a comprehensive overview of its diagnostic performance. Moreover, by focusing on studies across diverse clinical settings, we account for variability in surgical contexts and patient populations, enhancing the generalizability of our findings.

**Fig. 8:**
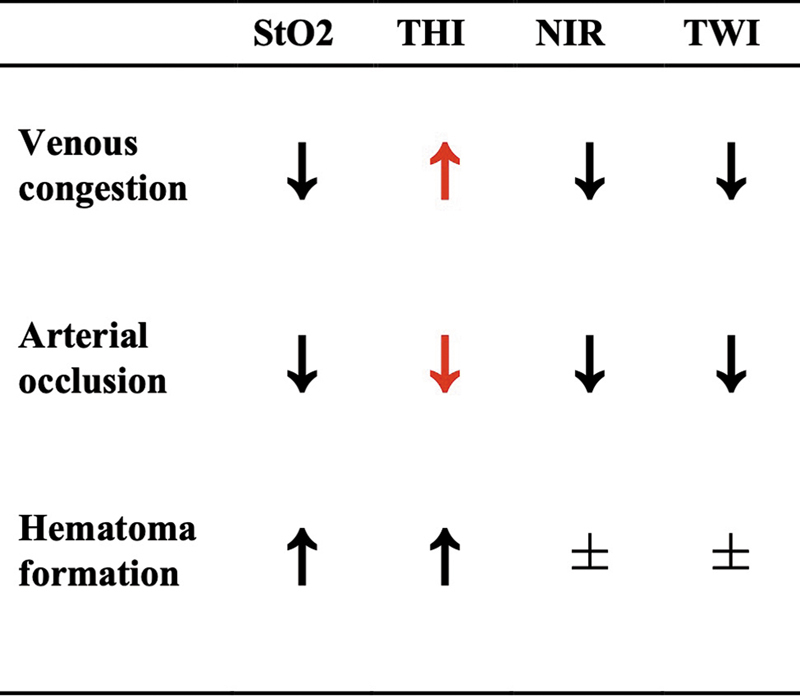
HSI parameters in a temporal context depending on the causes of malperfusion. Adapted from Schulz et al. (2020). 14 HSI, hyperspectral imaging; near-infrared perfusion index; StO _2_ , oxygen saturation; THI, tissue hemoglobin index; TWI, tissue water index.

However, several limitations must be acknowledged. The small sample sizes and observational designs of the included studies limit the statistical power of our meta-analysis. In particular, our analysis did not delve into the applicability of HSI for the detection of arterial occlusion and other malperfusion causes due to the lack of reported cases and statistical significance. For a high-level overview of the clinically expected temporal behavior of HSI parameters for venous congestion, arterial occlusion, and hematoma formation, see Fig. 8 . Furthermore, since we extracted data from graphs from the studies covered to analyze and compare HSI parameters, a difficult-to-quantify level of uncertainty and variation in precision were introduced in this analysis.

Significant heterogeneity in imaging protocols, device configurations, and patient characteristics poses challenges to establishing universal diagnostic thresholds. To address concerns about potential case overlaps in the studies by Schulz et al. (2020, 2021, and 2023), a sensitivity analysis was conducted (results not shown). We excluded Schulz et al. (2020), and subsequently also Schulz et al. (2020 and 2021), from the meta-analysis. In both cases, the statistical significance and direction of the findings for all HSI parameters remained unchanged. Also, the median diagnostic sensitivity rose to 97%, and specificity to 98%. Although explicit information on patient duplication was not available from the original publications, these robustness checks suggest that the results are duly influenced by potential case overlap. Finally, while HSI excels in intraoperative and postoperative monitoring, its discontinuous nature precludes real-time assessment, a key advantage of NIRS.

The findings of this review emphasize the importance of integrating HSI into routine flap monitoring to enhance early detection of complications and improve surgical outcomes. However, to fully realize its potential, future research should focus on

- Standardization: Developing consensus guidelines for HSI parameter thresholds and imaging protocols to reduce variability.
- Technology integration: Advancing real-time data processing capabilities for continuous monitoring.
- Cost-effectiveness analysis: Evaluating the economic impact of HSI implementation compared with traditional methods.
- Larger, multicenter trials: Conducting randomized controlled trials to validate its diagnostic accuracy and utility in diverse surgical contexts.

## Conclusion

HSI represents a promising innovation in flap monitoring, offering detailed, non-invasive insights into tissue viability. While our findings reinforce its diagnostic accuracy and versatility, addressing current limitations through standardization and further research will be critical for its widespread adoption. Complementing existing methods like NIRS, HSI stands poised to enhance outcomes in reconstructive surgery by enabling timely, data-driven interventions.
